# Midwife empathy and its association with the childbirth experience: a cross-sectional study

**DOI:** 10.1186/s12884-022-05309-3

**Published:** 2022-12-22

**Authors:** Yuhua Jin, Yanpeng Wu, Jing Li

**Affiliations:** 1 Department of Obstetrics and Gynecology, Shenzhen Samii Medical Center, Shenzhen, 518118 Guangdong Province China; 2 School of Public Health, Fudan University, Shanghai, 200433 China; 3 Department of Obstetrics and Gynecology, Shenzhen Hospital, Southern Medical University, Shenzhen, 518100 Guangdong Province China; 4 School of Nursing, Philippine Women’s University, 1743 Taft Avenue, 1004 Malate, Manila, Philippines

**Keywords:** Empathy, Chinese midwife, Jefferson scale of empathy, Childbirth

## Abstract

**Background:**

Although pregnancy and childbirth comprise a life-course that most midwives experience, whether their own experiences of childbirth resonate with other women during childbirth remains to be determined. In this study, we therefore characterized midwives’ empathic capabilities and defined their underlying factors.

**Method:**

We conducted a cross-sectional study with data from 464 midwives in Guangdong, China, that were collected through the “Chinese version of the Jefferson Scale of Empathy for Health Professionals (JSE-HP).” This questionnaire contains sections related to midwife demographics and delivery characteristics. We then implemented multivariate logistic regression analysis to identify empathy-related factors.

**Results:**

Our analysis revealed 303 (65.3%) participants in the high-empathy group while 161 (34.7%) were in the middle-empathy group. Compared with the reference groups, these results indicated that higher empathy was associated with an elevated educational level (OR, 1.83; 95% CI, 1.04–3.25), high monthly salary (OR, 2.30; 95% CI, 1.11–4.80), and no shift work (OR, 2.89; 95% CI, 1.09–7.63). The odds of a high empathy score were higher for midwives who experienced two childbirths (2.27, 1.11–4.66) and for those who had children under the age of 3 years (2.81, 1.34–5.92).

**Conclusion:**

Midwives possess a moderate-to-high level of empathy, and the greater the number of childbirths they experienced and the younger their children, the higher their reported empathy score. This study contributes novel information regarding the empathic behavior of midwives toward women who give birth in China.

**Supplementary Information:**

The online version contains supplementary material available at 10.1186/s12884-022-05309-3.

## Background

Midwifery is a concept that defines the care given to women during birth and is a function of the attending midwives [1]. Midwives play a crucial role in providing physical, psychological, and spiritual care to healthy women during childbirth and have been referred to as the “backbone” of maternal and newborn care [2, 3]. For example, midwives provide individualized education and antenatal care to women, observe labor progress, administer pain relief, perform appropriate midwifery techniques during labor, use uterine agents during the third stage of labor to prevent postpartum hemorrhage, and introduce early essential breastfeeding [4–6]. Such care requires competency in the child-birthing domain and requires the use of soft skills such as empathy [2]. Empathy is highly consistent with the ideologies of “humanistic care,” “psychological nursing,” or “high-quality nursing,” and has thus become one of the core concepts in the field of modern nursing [7]. There are even suggestions to add empathy as a core competency for a registered midwife [2].

In addition, maternal mental health problems are widespread around the world. A key recommendation for addressing the challenges of perinatal mental health is to integrate mental and maternal healthcare [8]. Improving the empathic engagement skills of maternity healthcare workers may enable them to respond more effectively to the mental health needs of their clients. Empathy professionals show patients extra respect and understanding, enabling them to actively express their true feelings. Careful listening by professionals and timely affirmation and empathy make it easier for patients to feel fully accepted and understood, and thus more receptive to additional treatments and interventions. In the process, a state of mutual trust is achieved between the medical staff and the patient. In addition, empathy can help patients ease their negative moods, enhance their confidence to overcome the disease, and makes them yearn for the wonderful things and fresh life in the future, so as to effectively improve the current situation [9]. Moloney et al. conducted a qualitative study and demonstrated that empathy contributed to the creation of a positive childbirth experience [10]. Owing to the revered nature of the birth process and its potential to be deeply imprinted into the mother’s psyche, a midwife’s lack of empathy and kindness coupled with disregard for the woman’s needs might contribute to a traumatizing experience for the mother. Such distress could then transcend into the period following birth [10, 11].

Previous data have shown a correlation between high levels of professional empathy and improved patient health outcomes, increased patient satisfaction, and diminished professional burnout [12]. However, there exist limited data on clinical midwives’ empathic levels or their effects on patient outcomes. In China, midwifery falls under the category of nursing, and graduates are required to pass a nursing licensing exam before they can be trained in clinical midwifery practice [13]. Since midwives principally care for pregnant and maternal women, the empathy levels of medical-surgical nurses do not necessarily reflect a complete representation of what is required by midwives, and the study of midwives is therefore of particular interest. Although pregnancy and childbirth constitute a life-course that most midwives experience, whether their own childbirth experience will resonate with other pregnant and puerperal women and engender different childbirth experiences and results is yet to be determined. In this study, we attempted to explain and characterize the empathic capabilities of midwives.

## Methods

### Study design and participants

The snowball sampling technique was used for recruitment through the WeChat group of Midwives’ Branch of the Guangdong Nurses Association (GNAM). WeChat is one of the most widely used social media platforms in China, featuring messaging, social communication and gaming features, especially during the COVID-19 pandemic. The members of the WeChat team are made up of head nurses or nursing leaders in the delivery rooms of major hospitals. First, the researchers pre-prepared the questionnaire and created a WeChat code or link entitled“A survey on the empathy ability of midwives in Guangdong Province”. An online questionnaire platform was used to compile the questionnaire, and the code or link was sent to committee members, who forwarded it to other midwives to fill out. The questionnaire was submitted only after all questions had been answered within a selected range of options. Only data from the full questionnaire was analyzed. Repeated entries are prevented by restricting multiple access to the survey by users with the same IP address. Midwives who were willing to voluntarily participate in the study, who had worked in the delivery room for at least 1 year as a midwife, and who held a certificate of technical service qualification in maternal and child health were included in our survey. Male midwives, licensed midwives not engaged in delivery room attendance, or midwives who were unable to independently complete the online questionnaire were excluded from the study.

The survey was open for data collection during May and June 2021. The sample size of variable influencing factor studies is mostly based on the requirements of statistical variable analysis, and it is recommended that the sample size should be at least 5–10 times of the number of variables [14]. This is a relatively simple way to estimate the sample size. In the study, the estimated sample size required was 190–380 based on 38 items (18 basic data items and 20 empathy scale items, see Supplementary file).

### Measurements

We applied the JSE-HP questionnaires to assess the degree of empathy developed by the midwives in the study. The English version of the JSE-HP was developed in 2001 by Dr. Mohammadreza Hojat and his research team at Thomas Jefferson University [15], and is currently one of the most frequently used questionnaires to assess the degree of empathy in healthcare. The JSE-HP has also been successfully translated into other languages (including Chinese), and has been shown to possess good reliability and validity for use by physicians, nurses, and other healthcare practitioners [16–18]. Similar to the original version, the Chinese JSE-HP is composed of 20 items on the 3 dimensions of perspective taking, compassionate care, and identifying with the patient; and each item is rated on a seven-point Likert scale. A total score between 20 and 140 points and higher indicated greater empathy. We calculated scores as score index = (the actual score on the scale/the highest possible score on the scale) × 100%. According to the score indices, the empathy scores were categorized at a low level (score < 56, 40%), moderate level (score = 56–112, 40–80%), or high level (score > 112, 80%). Demographic and sociologic characteristics such as age, educational level, monthly salary, shift work, marital status, mode of childbirth, and parity were collected using the questionnaire.

### Statistical analysis

We performed statistical analyses using R statistical software (version 4.0.3). Descriptive statistics were analyzed to describe the following features of the participants: socio-demographic characteristics, professional characteristics, and childbirth characteristics. The Chi-squared test was used to determine possible variables that were associated with empathy levels, and multivariate logistic regression analysis was executed to identify empathy-specific independent factors. Odds ratios (ORs) and 95% CIs were calculated for the variables in the ultimate equation.

## Results

The unit of analysis was 468 responses to the online survey (591 eligible midwifery members from 23 hospitals received links or codes from WeChat), excluding data from 4 identical or regular responses. A total of 464 valid questionnaires were collected, with a valid response rate of 99.1%, and we then compared the characteristics of the participants’ empathic abilities. Based on score index, the midwives’ overall scores for empathy were divided into two groups, high and middle (Table 1, Section 1). We observed that 303 (65.3%) participants were in the high-empathy group while 161 (34.7%) were in the middle-empathy group. Over 40% (198, 42.7%) of midwives had no childbirth experience themselves, 162 (34.9%) experienced a single childbirth, and 104 (22.4%) midwives underwent 2 childbirths. Of 266 midwives, 221 (83.1%) who experienced childbirth encountered labor pains; and 88 (39.8%) of these chose non-pharmacologic methods to reduce their labor pains, while 75 (33.9%) chose pharmacologic methods. The proportions of the midwives’ children who were at the three stages of infant, preschool, and school age were 35, 33.5, and 31.6%, respectively. Further analysis revealed that the midwives who were formal staff; or with a senior technical title, with educational levels of a bachelor’s or master’s degree, with a monthly salary of over 10,000 (CNY), without shift work, who were married, who showed a parity of two, and with children aged < 3 years were associated with a higher empathy level.

**Table 1:** Socio-demographic, professional and childbirth characteristics between respondents

Characteristics	Total (*N* = 464)	Section 1: Empathy levels			Section 2: Parity			
		high (*N* = 303)	middle (*N* = 161)	***p*** Value	0 (*N* = 198)	1 (*N* = 162)	2 (*N* = 104)	***p*** Value
**Age (years)**				0.237				**< 0.001**
<30	218 (47.0%)	134 (44.2%)	84 (52.2%)		166 (83.8%)	43 (26.5%)	9 (8.7%)	
30–40	187 (40.3%)	127 (41.9%)	60 (37.3%)		30 (15.2%)	72 (44.4%)	85 (81.7%)	
≥ 41	59 (12.7%)	42 (13.9%)	17 (10.6%)		2 (1.0%)	47 (29.0%)	10 (9.6%)	
**Technical title**				**0.045**				**< 0.001**
Junior	116 (25.0%)	71 (23.4%)	45 (28.0%)		94 (47.5%)	18 (11.1%)	4 (3.8%)	
Intermediate	180 (38.8%)	110 (36.3%)	70 (43.5%)		87 (43.9%)	56 (34.6%)	37 (35.6%)	
Senior	168 (36.2%)	122 (40.3%)	46 (28.6%)		17 (8.6%)	88 (54.3%)	63 (60.6%)	
**Educational level**				**0.013**				**< 0.001**
College degree or less	118 (25.4%)	66 (21.8%)	52 (32.3%)		81 (40.9%)	24 (14.8%)	13 (12.5%)	
Bachelor / master degree	346 (74.6%)	237 (78.2%)	109 (67.7%)		117 (59.1%)	138 (85.2%)	91 (87.5%)	
**Years of work**				0.171				**< 0.001**
1–5	176 (37.9%)	106 (35.0%)	70 (43.5%)		137 (69.2%)	28 (17.3%)	11 (10.6%)	
6–10	129 (27.8%)	86 (28.4%)	43 (26.7%)		46 (23.2%)	51 (31.5%)	32 (30.8%)	
≥ 11	159 (34.3%)	111 (36.6%)	48 (29.8%)		15 (7.6%)	83 (51.2%)	61 (58.7%)	
**Employment type**				0.080				**< 0.001**
Contract staff	319 (68.8%)	200 (66.0%)	119 (73.9%)		172 (86.9%)	88 (54.3%)	59 (56.7%)	
Formal staff	145 (31.2%)	103 (34.0%)	42 (26.1%)		26 (13.1%)	74 (45.7%)	45 (43.3%)	
**Monthly salary (CNY)**				**0.010**				**< 0.001**
< 5000	63 (13.6%)	34 (11.2%)	29 (18.0%)		47 (23.7%)	7 (4.3%)	9 (8.7%)	
5000-10,000	232 (50.0%)	145 (47.9%)	87 (54.0%)		103 (52.0%)	79 (48.8%)	50 (48.1%)	
>10,000	169 (36.4%)	124 (40.9%)	45 (28.0%)		48 (24.2%)	76 (46.9%)	45 (43.3%)	
**Shift work**				**0.017**				**< 0.001**
No	40 (8.6%)	33 (10.9%)	7 (4.3%)		1 (0.5%)	28 (17.3%)	11 (10.6%)	
Yes	424 (91.4%)	270 (89.1%)	154 (95.7%)		197 (99.5%)	134 (82.7%)	93 (89.4%)	
**Marital status**				**0.048**				**< 0.001**
Single	154 (33.2%)	91 (30.0%)	63 (39.1%)		154 (77.8%)	0 (0.0%)	0 (0.0%)	
Married	310 (66.8%)	212 (70.0%)	98 (60.9%)		44 (22.2%)	162 (100.0%)	104 (100.0%)	
**Abortion history (times)**				0.975				**< 0.001**
0	352 (75.9%)	230 (75.9%)	122 (75.8%)		190 (96.0%)	97 (59.9%)	65 (62.5%)	
1	112 (24.1%)	73 (24.1%)	39 (24.2%)		8 (4.0%)	65 (40.1%)	39 (37.5%)	
**Parity**				**0.011**	–	–	–	–
0	198 (42.7%)	118 (38.9%)	80 (49.7%)		–	–	–	
1	162 (34.9%)	105 (34.7%)	57 (35.4%)		–	–	–	
2	104 (22.4%)	80 (26.4%)	24 (14.9%)		–	–	–	
**Mode of childbirth**				0.244				0.093
N-Miss	198	118	80					
Vaginal delivery	165 (62.0%)	119 (64.3%)	46 (56.8%)		–	94 (58.0%)	71 (68.3%)	
Cesarean section	101 (38.0%)	66 (35.7%)	35 (43.2%)		–	68 (42.0%)	33 (31.7%)	
**Labor pain experience**				0.414				**0.027**
N-Miss	198	118	80					
No	45 (16.9%)	29 (15.7%)	16 (19.8%)		–	34 (21.0%)	11 (10.6%)	
Yes	221 (83.1%)	156 (84.3%)	65 (80.2%)		–	128 (79.0%)	93 (89.4%)	
**Pain relief method**				0.347				**0.012**
N-Miss	243	147	96		–	34	11	
No	58 (26.2%)	37 (23.7%)	21 (32.3%)		–	41 (32.0%)	17 (18.3%)	
Non-pharmacological	88 (39.8%)	66 (42.3%)	22 (33.8%)		–	41 (32.0%)	47 (50.5%)	
Pharmacological	75 (33.9%)	53 (34.0%)	22 (33.8%)		–	46 (35.9%)	29 (31.2%)	
**Labor complications**				0.065				0.326
N-Miss	198	118	80					
No	248 (93.2%)	169 (91.4%)	79 (97.5%)		–	153 (94.4%)	95 (91.3%)	
Yes	18 (6.8%)	16 (8.6%)	2 (2.5%)		–	9 (5.6%)	9 (8.7%)	
**Children age (years)**				**0.033**				**< 0.001**
N-Miss	198	118	80					
Infant (<3)	93 (35.0%)	73 (39.5%)	20 (24.7%)		–	48 (29.6%)	45 (43.3%)	
Preschool (3–5)	89 (33.5%)	54 (29.2%)	35 (43.2%)		–	41 (25.3%)	48 (46.2%)	
School-age (≥6)	84 (31.6%)	58 (31.4%)	26 (32.1%)		–	73 (45.1%)	11 (10.6%)	

In addition, a comparison of childbirth characteristics showed that most of the variables were significantly related to the number of childbirths (Table 1, section 2). A majority of midwives with two deliveries were more likely to have achieved a higher educational level, exhibit more years of work, possess a higher professional title, and be between 30 and 40 years of age. Regardless of childbirth experience, over 80% of the midwives worked night shifts.

We then determined the correlation between empathy levels and the characteristics of the 464 midwives using a stepwise logistic regression analysis (Table 2). Based on the potential variables reported in previous studies [18–21], the actual situation in China, and the result of a single-factor analysis, we selected parity, age, technical title, educational level, years of work, employment type, monthly salary, and shift work as variables for the multivariate logistic regression. Our results indicated that midwives who had undergone two childbirths manifested more than twice the level of empathy compared to midwives who had no delivery experience (OR, 2.35; 95% CI, 1.18–4.69), while midwives with a bachelor’s or master’s degree were over twice as likely to possess a high-empathy level relative to those with a college degree or less (1.83, 1.04–3.25). The midwives with a high monthly salary (≥ 10,000 CNY) exhibited a greater likelihood of showing a high-empathy level compared to those with a low monthly salary (< 5000 CNY) (2.30, 1.11–4.80), while no shift work performed by midwives was associated with a higher empathy level as indicated by an OR of 2.89 (1.09–7.63) (Table 2, Model 2). In the multiple logistic regression model encompassing 266 midwife deliveries, we included variables such as parity, age, technical title, educational level, years of work, employment type, monthly salary, shift work, mode of childbirth, labor pain experience, and children’s age (Table 3). This model revealed that midwives who had undergone two childbirths were twice as likely to report a high level of empathy compared to those who had one childbirth experience (2.27, 1.11–4.66); and that midwives who themselves had children at the infant stage (< 3 years of age) showed twice the odds of reporting a high level of empathy relative to those who had children in preschool (3–5-year-olds) (2.81, 1.34–5.92) (Table 3, Model 5).

**Table 2:** Multiple logistic regression model for all women including baseline characteristic

Variable	Model 1		Model 2	
	OR (95% CI)	***p*** Value	OR (95% CI)	***p*** Value
**Parity**				
0	1(ref)		1(ref)	
1	1.25 (0.81, 1.92)	0.31	1.05 (0.60, 1.84)	0.86
2	2.26 (1.32, 3.87)	0.003	2.35 (1.18, 4.69)	**0.02**
**Age (years)**				
<30			1(ref)	
30–40			0.68 (0.34, 1.37)	0.28
>41			0.61 (0.21, 1.80)	0.37
**Technical title**				
Junior			1(ref)	
Intermediate			0.51 (0.26, 1.01)	0.05
Senior			0.69 (0.26, 1.80)	0.44
**Educational level**				
College degree or less			1(ref)	
Bachelor/master degree			1.83 (1.04, 3.25)	**0.04**
**Years of work (years)**				
1–5			1(ref)	
6–10			1.17 (0.64, 2.13)	0.61
≥ 11			0.96 (0.43, 2.15)	0.91
**Employment type**				
Contract staff			1(ref)	
Formal staff			1.17 (0.70, 1.95)	0.55
**Monthly salary (CNY)**				
< 5000			1(ref)	
5000-10,000			1.39 (0.75, 2.59)	0.30
>10,000			2.30 (1.11, 4.80)	**0.03**
**Shift work**				
No			1(ref)	
Yes			2.89 (1.09, 7.63)	**0.03**

*OR* Odds ratio, *CI* confidence interval, *ref* reference category

**Table 3:** Multiple logistic regression model for delivered women including variables related to delivery record

Variable	Model 3		Model 4		Model 5	
	OR (95% CI)	***p*** Value	OR (95% CI)	***p*** Value	OR (95% CI)	*p* Value
**Parity**						
1			1(ref)		1(ref)	
2	1.81 (1.04, 3.16)	0.04	2.39 (1.26, 4.51)	0.01	2.27 (1.11, 4.66)	**0.03**
**Age (years)**						
>41			1(ref)		1(ref)	
<30			2.92 (0.82, 10.45)	0.10	2.07 (0.52, 8.23)	0.30
30–40			1.13 (0.48, 2.67)	0.78	0.99 (0.39, 2.50)	0.98
**Technical title**						
Junior			1(ref)		1(ref)	
Intermediate			0.76 (0.25, 2.27)	0.62	0.96 (0.31, 3.00)	0.94
Senior			1.37 (0.37, 5.07)	0.64	1.77 (0.45, 6.91)	0.41
**Educational level**						
College degree or less			1(ref)		1(ref)	
Bachelor/master degree			2.03 (0.90, 4.59)	0.09	2.12 (0.90, 4.98)	0.09
**Years of work (years)**						
1–5			1(ref)		1(ref)	
6–10			1.67 (0.72, 3.90)	0.24	1.82 (0.76, 4.37)	0.18
≥ 11			1.23 (0.46, 3.29)	0.68	1.43 (0.51, 4.02)	0.50
**Employment type**						
Contract staff			1(ref)		1(ref)	
Formal staff			1.09 (0.57, 2.06)	0.80	1.07 (0.54, 2.10)	0.86
**Monthly salary (CNY)**						
< 5000			1(ref)		1(ref)	
5000-10,000			1.14 (0.36, 3.68)	0.82	1.21 (0.37, 3.98)	0.76
>10,000			2.31 (0.66, 8.05)	0.19	2.62 (0.73, 9.37)	0.14
**Shift work**						
No			1(ref)		1(ref)	
Yes			2.84 (1.02, 7.92)	0.05	3.03 (1.07, 8.57)	**0.04**
**Mode of childbirth**						
Vaginal delivery					1(ref)	
Cesarean section					0.63 (0.31, 1.28)	0.20
**Labor pain experience**						
Yes					1(ref)	
No					0.79 (0.32, 1.94)	0.61
**Perinatal depression**						
Yes					1(ref)	
No					0.94 (0.51, 1.73)	0.83
**Children age (years)**						
Preschool (3–5)					1(ref)	
Infant (<3)					2.81 (1.34, 5.92)	**0.01**
School-age (≥6)					1.18 (0.44, 3.16)	0.74

*OR* Odds ratio, *CI* confidence interval, *ref* reference category

## Discussion

This is the first-ever investigation of the empathy ability of midwives in China, and our findings form the basis for future research. In the present study, 65.3% of the midwives met the high-empathy level while the remainder professed middle-empathy levels. The effects of the midwives’ childbirth experiences on their empathy levels was a highlight of this study, as we found that the number of births experienced by midwives and the age of their children were highly correlated with high levels of empathy.

Our results suggested that midwives who underwent two deliveries themselves had a two-fold higher level of empathy than those with fewer personal delivery experiences. These data were consistent with those of previous studies [22–25] that showed that midwives who had undergone labor pain were more likely to demonstrate more empathy to the laboring women under their care; and that empathy was built upon shared experiences and connections [26]. Midwives who had experience in childbirth were willing to share their own experiences, and provided guidance, encouragement, and support to other women. This interactive approach creates a positive atmosphere for puerpera, promoting sufficient progress in labor.

However, our results depicted no significant difference in empathy between midwives who gave birth once and those who did not, and this was consistent with earlier findings on empathy measures and estimates of patient pain [23]. These authors demonstrated that midwives’ personal characteristics could skew the estimation of pain in woman in labor. For example, a midwife who manifested abundant personal childbirth experience estimated labor pain as higher, while pain estimates were even higher for midwives who had elevated scores on empathy. This suggested to us that empathy for the laboring woman could be effectively targeted, but that it might also be related to labor analgesia. Epidural analgesia is known to be a highly effective way to improve comfort in women, and this might affect the stimulation of the midwives’ empathic abilities to an extent. In addition, it is a common phenomenon that women with a parity of two or more are less likely to receive epidural analgesia than uniparas; and this might constitute one of the reasons as to why midwives, doctors, and anesthesiologists posit that multiparas require lower pain relief due to their shorter labor duration [27]. Thus, midwives who bore two children might suffer greater labor pain. In addition, “memory of labor pain” is another intriguing issue with respect to in-labor pain intensity at an individual level [27]. A study of long-term memory of labor pain showed that women’s recollections of labor pain intensity changed over time. However, a significant minority remembered the pain as more intense at 5 years than at 2 months postpartum. A positive overall birth experience was associated with a memory of less pain over time, but this was not the case for women with negative birth experiences. In addition, higher pain scores might have been expected at the 5-year assessment in women who had another baby after the index birth, since the time elapsed after re-experiencing labor pain was shorter. Evaluation of the birth experience may have implications for future reproduction. Women who remembered childbirth as a negative experience had fewer subsequent children and a longer gap between their next pregnancies than those who had a positive overall experience. Fear of pain is one of the reasons given by pregnant women who worry about the approaching birth or request a caesarean section [28].

Our results indicated that the age of the midwives’ children impacted empathy levels significantly, as midwives who had children at the infant stage (< 3 years) exhibited higher levels of empathy. We hypothesize that this could be due to the fact that parental care is an ancient root form from which more complex forms of empathy have emerged [29, 30]. Hodges et al. compared the empathy of new mothers, non-mothers, and pregnant women, and showed the greatest empathy and understanding in new mothers [22]. Immediately after childbirth and during the first few neonatal months, mothers as the primary caretakers of young infants often synchronize their own biologic cycle and daily activities with the infants’ physical and psychological needs [31]. Empathy might then be heightened in parents as it favors caregiving and the perception of non-verbal needs from infants, as well as increased infant survival [31, 32]. Multiple studies have, for example, shown the effects of oxytocin in promoting empathetic abilities [29, 30, 33, 34]. Oxytocin is an endogenous neuropeptide associated with bonding and nurturing behavior, and oxytocin is therefore considered to be a robust mediator of empathy. This concept stems from observations of the beneficial effects of intranasal oxytocin on cognitive aspects of empathy—including the processing of social information, mind reading, and emotion recognition [33, 35, 36]. During pregnancy and the postpartum period, hormones such as prolactin, oxytocin, and progesterone increase and prepare the body for childbirth, nursing, and child rearing [37, 38]. Also previous investigators have proposed that pregnancy and the postpartum period mediate an elevation in maternal sensitivity [37, 39]. These changes affect how women respond to emotional information in their surrounding environment and shape their behaviors in response to exclusive stimuli in motherhood or mother-baby interactions [37]. The sensitivity used by parents to tailor their behaviors to those of their immature offspring is not only found within the mother–infant relationship, but also with respect to other group members [30, 40].

Based on our multivariate logistic analysis, similar independent variables were associated with empathy; for instance, shift work was associated with a lower empathy level. These findings were congruent with previous evidence showing that sleep deprivation and mood disorders exerted a detrimental impact on cognitive processing and might reduce a persons’ capacity for empathy [32, 41, 42]. Our research showed that over 80% of midwives worked night shifts. The result may be due to the nature of midwifery. The heavy workload at night is one of the characteristics of the delivery room. Data showed that labor commenced more regularly in night shift, accounting for 53.7% [43]. This may be due to the elevated number of women who underwent induction of labor by oxytocin drip during the day shift, and these women were more likely to enter labor during the night shift [43]. In addition, researchers documented that in all cases more than 36 weeks gestation the oxytocin levels were higher at night-time [44]. It was possible for multiple women to give birth at night at the same time. The midwifery work is also characterized by strong randomness, various emergencies and high rescue risk. In order to ensure the safety, adequate midwives are required, and they need to be “on call”, which is the greatest hindrance to sleep. Sleep deprivation thus exerts a negative impact on a midwife’s individual health and well-being, as well as on the health of the woman under the midwife’s care. After the COVID-19 outbreak, midwives, as other healthcare workers, have been working longer hours and/or with different shift patterns, and reassigned to the front lines to fight the virus [45]. Midwives face increasing work pressure and low levels of personal fulfillment, which increases their likelihood of developing severe job burnout. This situation has led to a sustained decline in empathy. Numerous strategies have been employed to increase the quality and amount of midwives’ sleep—including schedule modifications, structural changes, and altered staffing ratios within their practices [1].

In order to improve the population structure and actively respond to the aging population, China’s three-child policy was implemented in May 2021 [46]. Even after the announcement of the three-child policy, the intention by couples to have a third child remained low in the general Chinese population. Conducted by state news agency Xinhua, of 31, 000 people polled, only 4–5% were ready to have a third child, and 90% said they would not consider it [47]. The Chinese government has thus taken a series of measures to encourage fertility—including raising maternity benefits, reducing the burden of parenting, and strengthening infant care services. However, these measures have had a limited effect. Coupled with this is the cultural legacy of the one-child policy, points out Yi Fuxian, a senior scientist in obstetrics and gynaecology at the University of Wisconsin-Madison (Madison, WI, USA). “The one-child policy has changed Chinese childbearing attitudes”, he points out. “Having only one child or no children has become the social norm in China. Social and economic patterns cater to the one-child policy, so inertial effects linger [47].” A systematic qualitative review of what matters to women during childbirth suggests that most women around the world hope for a positive labor and birth experience that enables them to give birth to a healthy baby in a clinically and psychologically safe environment with practical and emotional support from birth companions, and competent, reassuring, kind clinical staff [48].

Influential departments in hospitals and society should pay more attention to improving the empathy of midwives as much as possible to ensure that maternal needs are met. First, empathy in midwifery should be part of the curriculum for every student to graduate with this perspective. It has been determined that humanities-based reflective practices, clinical simulation, role modeling, and contemplative practice are ideal teaching methods to gain compassion in clinical education [44]. Second, hospitals should create a respectful, harmonious and warm work atmosphere. Improving the working environment for midwives could be an effective way to boost their compassion and satisfaction [49]. Finally, midwifery leaders and health policy makers should be sensitive to the issue of manpower shortages. The number of pregnant women per midwife should be decreased to facilitate women’s/families’ access to prenatal care and the word ‘empathy’ should be included in health strategy, policy, employment and training processes [21].

## Limitations

There were limitations to the present study. First, the data was collected using a self-administered web-based questionnaire via smartphones, which may have resulted in information bias and misclassification bias. It is possible that participants did not provide accurate information in order to either be included in the study or to move quickly through the survey. Second, the online study used only self-reported measures and did not consider patient opinion. Thus, it is a necessity to triangulate the information to increase data reliability, using all measures, professionals, patients, and outside observers [50]. In the future, a validated instrument that measures empathy of health professionals as viewed from the patients’ perspective is required to investigate whether an overlap is found between self-reporting and patient-reporting scores of empathy [51]. Finally, the results from this study might not be generalizable to all Chinese midwives, as we only collected samples from Guangdong province; the data are, therefore, not representative of all midwives in China. More studies are required to determine whether empathy is enhanced or suppressed during the important transition from a girl’s adolescent period to motherhood. Thus, replication of our research on midwives is a necessity for other provinces and countries.

## Conclusions

Our data collectively showed that the experience of childbirth exerted a positive impact on midwife empathy. Midwives are not only providers of childbirth services, but are also recipients as well. A positive childbirth experience will, in turn, affects the midwife’s empathy for her client—i.e., the woman giving birth—and thus affect the client’s overall birth experience. This phenomenon constitutes a “virtuous circle,” and we speculate that enhancing the empathy level of midwives will improve women’s willingness to give birth. These findings therefore contribute significantly to refining our approach to the provision of improved perinatal care.

## Supplementary Information

**Additional file 1.**

## Data Availability

All data generated or analysed during this study are included in this published article and its supplementary information files.

## Abbreviations

JSE-HP: Jefferson Scale of Empathy for Health Professionals
OR: Odds ratios
CI: Confidence interval
CNY: China Yuan
