# Supplementary material for: Midwife empathy and its association with the childbirth experience: a cross-sectional study
Source: BMC Pregnancy Childbirth. 2022 Dec 22;22:960. doi: 10.1186/s12884-022-05309-3 (PMC9774080; doi:10.1186/s12884-022-05309-3)
Supplement: Supplementary file 1 — Additional file 1. [file 12884_2022_5309_MOESM1_ESM.zip › Supplementary file/Statistical analysis results.docx]

Statistical analysis results

**Table 1. Demographics and professional characteristics between respondents with high empathy scores and middle scores**

| **Variables** | **high (N=303)** | **middle (N=161)** | **Total (N=464)** | **p value** |
| --- | --- | --- | --- | --- |
| **Hospital grade** |  |  |  | 0.688 |
| Tertiary hospital (grade A) | 233 (76.9%) | 116 (72.0%) | 349 (75.2%) |  |
| Tertiary hospital (grade B) | 11 (3.6%) | 6 (3.7%) | 17 (3.7%) |  |
| Secondary hospital (grade A) | 54 (17.8%) | 36 (22.4%) | 90 (19.4%) |  |
| Secondary hospital (grade B) | 5 (1.7%) | 3 (1.9%) | 8 (1.7%) |  |
| **Age （years）** |  |  |  | 0.237 |
| ＜30 | 134 (44.2%) | 84 (52.2%) | 218 (47.0%) |  |
| 30-40 | 127 (41.9%) | 60 (37.3%) | 187 (40.3%) |  |
| ≥41 | 42 (13.9%) | 17 (10.6%) | 59 (12.7%) |  |
| **Technical title** |  |  |  | 0.045 |
| Junior | 71 (23.4%) | 45 (28.0%) | 116 (25.0%) |  |
| Intermediate | 110 (36.3%) | 70 (43.5%) | 180 (38.8%) |  |
| Senior | 122 (40.3%) | 46 (28.6%) | 168 (36.2%) |  |
| **Educational level** |  |  |  | 0.013 |
| College degree or less | 66 (21.8%) | 52 (32.3%) | 118 (25.4%) |  |
| Bachelor / master degree | 237 (78.2%) | 109 (67.7%) | 346 (74.6%) |  |
| **Years of work** |  |  |  | 0.171 |
| 1-5 | 106 (35.0%) | 70 (43.5%) | 176 (37.9%) |  |
| 6-10 | 86 (28.4%) | 43 (26.7%) | 129 (27.8%) |  |
| ≥11 | 111 (36.6%) | 48 (29.8%) | 159 (34.3%) |  |
| **Employment type** |  |  |  | 0.080 |
| Contract staff | 200 (66.0%) | 119 (73.9%) | 319 (68.8%) |  |
| Formal staff | 103 (34.0%) | 42 (26.1%) | 145 (31.2%) |  |
| **Monthly salary (CNY)** |  |  |  | 0.010 |
| ＜ 5,000 | 34 (11.2%) | 29 (18.0%) | 63 (13.6%) |  |
| 5,000-10,000 | 145 (47.9%) | 87 (54.0%) | 232 (50.0%) |  |
| ＞10,000 | 124 (40.9%) | 45 (28.0%) | 169 (36.4%) |  |
| **Shift work** |  |  |  | 0.017 |
| No | 33 (10.9%) | 7 (4.3%) | 40 (8.6%) |  |
| Yes | 270 (89.1%) | 154 (95.7%) | 424 (91.4%) |  |
| **Marital status** |  |  |  | 0.048 |
| Single | 91 (30.0%) | 63 (39.1%) | 154 (33.2%) |  |
| Married | 212 (70.0%) | 98 (60.9%) | 310 (66.8%) |  |
| **Abortion history** |  |  |  | 0.975 |
| 0 | 230 (75.9%) | 122 (75.8%) | 352 (75.9%) |  |
| 1 | 73 (24.1%) | 39 (24.2%) | 112 (24.1%) |  |
| **Parity** |  |  |  | 0.011 |
| 0 | 118 (38.9%) | 80 (49.7%) | 198 (42.7%) |  |
| 1 | 105 (34.7%) | 57 (35.4%) | 162 (34.9%) |  |
| 2 | 80 (26.4%) | 24 (14.9%) | 104 (22.4%) |  |
| **Mode of childbirth** |  |  |  | 0.244 |
| N-Miss | 118 | 80 | 198 |  |
| Vaginal delivery | 119 (64.3%) | 46 (56.8%) | 165 (62.0%) |  |
| Cesarean section | 66 (35.7%) | 35 (43.2%) | 101 (38.0%) |  |
| **Labor pain experience** |  |  |  | 0.414 |
| N-Miss | 118 | 80 | 198 |  |
| No | 29 (15.7%) | 16 (19.8%) | 45 (16.9%) |  |
| Yes | 156 (84.3%) | 65 (80.2%) | 221 (83.1%) |  |
| **Pain relief method** |  |  |  | 0.347 |
| N-Miss | 147 | 96 | 243 |  |
| No | 37 (23.7%) | 21 (32.3%) | 58 (26.2%) |  |
| Non-pharmacological | 66 (42.3%) | 22 (33.8%) | 88 (39.8%) |  |
| Pharmacological | 53 (34.0%) | 22 (33.8%) | 75 (33.9%) |  |
| **Labor complications** |  |  |  | 0.065 |
| N-Miss | 118 | 80 | 198 |  |
| No | 169 (91.4%) | 79 (97.5%) | 248 (93.2%) |  |
| Yes | 16 (8.6%) | 2 (2.5%) | 18 (6.8%) |  |
| **Perinatal depression** |  |  |  | 0.520 |
| N-Miss | 118 | 80 | 198 |  |
| No | 124 (67.0%) | 51 (63.0%) | 175 (65.8%) |  |
| Yes | 61 (33.0%) | 30 (37.0%) | 91 (34.2%) |  |
| **Children age （years）** |  |  |  | 0.033 |
| N-Miss | 118 | 80 | 198 |  |
| Infant (＜3) | 73 (39.5%) | 20 (24.7%) | 93 (35.0%) |  |
| Preschool (3-5) | 54 (29.2%) | 35 (43.2%) | 89 (33.5%) |  |
| School-age (≥6) | 58 (31.4%) | 26 (32.1%) | 84 (31.6%) |  |

**Table 2. Demographics and professional characteristics among respondents with** **different delivery times**

| **Variables** | 0 (N=198) | 1 (N=162) | 2 (N=104) | Total (N=464) | p value |
| --- | --- | --- | --- | --- | --- |
| **Empathy level** |  |  |  |  | 0.011 |
| high | 118 (59.6%) | 105 (64.8%) | 80 (76.9%) | 303 (65.3%) |  |
| middle | 80 (40.4%) | 57 (35.2%) | 24 (23.1%) | 161 (34.7%) |  |
| **Hospital grade** |  |  |  |  | 0.042 |
| Tertiary hospital (grade A) | 156 (78.8%) | 125 (77.2%) | 68 (65.4%) | 349 (75.2%) |  |
| Tertiary hospital (grade B) | 9 (4.5%) | 3 (1.9%) | 5 (4.8%) | 17 (3.7%) |  |
| Secondary hospital (grade A) | 31 (15.7%) | 29 (17.9%) | 30 (28.8%) | 90 (19.4%) |  |
| Secondary hospital (grade B) | 2 (1.0%) | 5 (3.1%) | 1 (1.0%) | 8 (1.7%) |  |
| **Age (years)** |  |  |  |  | < 0.001 |
| ＜30 | 166 (83.8%) | 43 (26.5%) | 9 (8.7%) | 218 (47.0%) |  |
| 30-40 | 30 (15.2%) | 72 (44.4%) | 85 (81.7%) | 187 (40.3%) |  |
| ≥41 | 2 (1.0%) | 47 (29.0%) | 10 (9.6%) | 59 (12.7%) |  |
| **Technical title** |  |  |  |  | < 0.001 |
| Junior | 94 (47.5%) | 18 (11.1%) | 4 (3.8%) | 116 (25.0%) |  |
| Intermediate | 87 (43.9%) | 56 (34.6%) | 37 (35.6%) | 180 (38.8%) |  |
| Senior | 17 (8.6%) | 88 (54.3%) | 63 (60.6%) | 168 (36.2%) |  |
| **Educational level** |  |  |  |  | < 0.001 |
| College degree or less | 81 (40.9%) | 24 (14.8%) | 13 (12.5%) | 118 (25.4%) |  |
| Bachelor / master degree | 117 (59.1%) | 138 (85.2%) | 91 (87.5%) | 346 (74.6%) |  |
| **Years of work** |  |  |  |  | < 0.001 |
| 1-5 | 137 (69.2%) | 28 (17.3%) | 11 (10.6%) | 176 (37.9%) |  |
| 6-10 | 46 (23.2%) | 51 (31.5%) | 32 (30.8%) | 129 (27.8%) |  |
| ≥11 | 15 (7.6%) | 83 (51.2%) | 61 (58.7%) | 159 (34.3%) |  |
| **Employment Type** |  |  |  |  | < 0.001 |
| Contract staff | 172 (86.9%) | 88 (54.3%) | 59 (56.7%) | 319 (68.8%) |  |
| Formal staff | 26 (13.1%) | 74 (45.7%) | 45 (43.3%) | 145 (31.2%) |  |
| **Monthly salary （CNY）** |  |  |  |  | < 0.001 |
| ＜ 5,000 | 47 (23.7%) | 7 (4.3%) | 9 (8.7%) | 63 (13.6%) |  |
| 5,000-10,000 | 103 (52.0%) | 79 (48.8%) | 50 (48.1%) | 232 (50.0%) |  |
| ＞10,000 | 48 (24.2%) | 76 (46.9%) | 45 (43.3%) | 169 (36.4%) |  |
| **Shift work** |  |  |  |  | < 0.001 |
| No | 1 (0.5%) | 28 (17.3%) | 11 (10.6%) | 40 (8.6%) |  |
| Yes | 197 (99.5%) | 134 (82.7%) | 93 (89.4%) | 424 (91.4%) |  |
| **Marital status** |  |  |  |  | < 0.001 |
| Single | 154 (77.8%) | 0 (0.0%) | 0 (0.0%) | 154 (33.2%) |  |
| Married | 44 (22.2%) | 162 (100.0%) | 104 (100.0%) | 310 (66.8%) |  |
| **Abortion history** |  |  |  |  | < 0.001 |
| 0 | 190 (96.0%) | 97 (59.9%) | 65 (62.5%) | 352 (75.9%) |  |
| 1 | 8 (4.0%) | 65 (40.1%) | 39 (37.5%) | 112 (24.1%) |  |
| **Mode of childbirth** |  |  |  |  | 0.093 |
| Vaginal delivery | — | 94 (58.0%) | 71 (68.3%) | 165 (62.0%) |  |
| Cesarean section | — | 68 (42.0%) | 33 (31.7%) | 101 (38.0%) |  |
| **Labor pain experience** |  |  |  |  | 0.027 |
| No | — | 34 (21.0%) | 11 (10.6%) | 45 (16.9%) |  |
| Yes | — | 128 (79.0%) | 93 (89.4%) | 221 (83.1%) |  |
| **Pain relief method** |  |  |  |  | 0.012 |
| N-Miss | — | 34 | 11 | 45 |  |
| No | — | 41 (32.0%) | 17 (18.3%) | 58 (26.2%) |  |
| Non-pharmacological | — | 41 (32.0%) | 47 (50.5%) | 88 (39.8%) |  |
| Pharmacological | — | 46 (35.9%) | 29 (31.2%) | 75 (33.9%) |  |
| **Labor complications** |  |  |  |  | 0.326 |
| No | — | 153 (94.4%) | 95 (91.3%) | 248 (93.2%) |  |
| Yes | — | 9 (5.6%) | 9 (8.7%) | 18 (6.8%) |  |
| **Perinatal depression** |  |  |  |  | 0.878 |
| No | — | 106 (65.4%) | 69 (66.3%) | 175 (65.8%) |  |
| Yes | — | 56 (34.6%) | 35 (33.7%) | 91 (34.2%) |  |
| **Children age （years）** |  |  |  |  | < 0.001 |
| Infant (＜3) | — | 48 (29.6%) | 45 (43.3%) | 93 (35.0%) |  |
| Preschool (3-5) | — | 41 (25.3%) | 48 (46.2%) | 89 (33.5%) |  |
| School-age (≥6) | — | 73 (45.1%) | 11 (10.6%) | 84 (31.6%) |  |

| **Table 3. Multiple logistic regression model for all women including baseline characteristic** | | | | |
| --- | --- | --- | --- | --- |
| **Variable** | **Model 1** | | **Model 2** | |
|  | OR (95% CI) | p value | OR (95% CI) | p value |
| **Childbirth times** |  |  |  |  |
| 0 | 1（ref） |  | 1（ref） |  |
| 1 | 1.25 (0.81, 1.92) | 0.31 | 1.05 (0.60, 1.84) | 0.86 |
| 2 | 2.26 (1.32, 3.87) | 0.003 | 2.35 (1.18, 4.69) | 0.02 |
| **Age （years）** |  |  |  |  |
| ＜30 |  |  | 1（ref） |  |
| 30-40 |  |  | 0.68 (0.34, 1.37) | 0.28 |
| ＞41 |  |  | 0.61 (0.21, 1.80) | 0.37 |
| **Technical title** |  |  |  |  |
| Junior |  |  | 1（ref） |  |
| Intermediate |  |  | 0.51 (0.26, 1.01) | 0.05 |
| Senior |  |  | 0.69 (0.26, 1.80) | 0.44 |
| **Educational level** |  |  |  |  |
| College degree or less |  |  | 1（ref） |  |
| Bachelor/master degree | |  | 1.83 (1.04, 3.25) | 0.04 |
| **Years of work** |  |  |  |  |
| 1-5 |  |  | 1（ref） |  |
| 6-10 |  |  | 1.17 (0.64, 2.13) | 0.61 |
| ≥11 |  |  | 0.96 (0.43, 2.15) | 0.91 |
| **Employment form** |  |  |  |  |
| Contract staff |  |  | 1（ref） |  |
| Formal staff |  |  | 1.17 (0.70, 1.95) | 0.55 |
| **Monthly salary （CNY）** |  |  |  |  |
| ＜ 5,000 |  |  | 1（ref） |  |
| 5,000-10,000 |  |  | 1.39 (0.75, 2.59) | 0.30 |
| ＞10,000 |  |  | 2.30 (1.11, 4.80) | 0.03 |
| **Shift work** |  |  |  |  |
| No |  |  | 1（ref） |  |
| Yes |  |  | 2.89 (1.09, 7.63) | 0.03 |

| **Table 4. Multiple logistic regression model for delivered women including variables related to delivery record** | | | | | | |
| --- | --- | --- | --- | --- | --- | --- |
| **Variable** | **Model 3** | | **Model 4** | | **Model 5** | |
|  | OR (95% CI) | p value | OR (95% CI) | p value | OR (95% CI) | p value |
| **Childbirth times** |  |  |  |  |  |  |
| 1 |  |  | 1(ref) |  | 1(ref) |  |
| 2 | 1.81 (1.04, 3.16) | 0.04 | 2.39 (1.26, 4.51) | 0.01 | 2.27 (1.11, 4.66) | 0.03 |
| **Age （years）** |  |  |  |  |  |  |
| ＞41 |  |  | 1(ref) |  | 1(ref) |  |
| ＜30 |  |  | 2.92 (0.82, 10.45) | 0.10 | 2.07 (0.52, 8.23) | 0.30 |
| 30-40 |  |  | 1.13 (0.48, 2.67) | 0.78 | 0.99 (0.39, 2.50) | 0.98 |
| **Technical title** |  |  |  |  |  |  |
| Junior |  |  | 1(ref) |  | 1(ref) |  |
| Intermediate |  |  | 0.76 (0.25, 2.27) | 0.62 | 0.96 (0.31, 3.00) | 0.94 |
| Senior |  |  | 1.37 (0.37, 5.07) | 0.64 | 1.77 (0.45, 6.91) | 0.41 |
| **Educational level** |  |  |  |  |  |  |
| College degree or less | |  | 1(ref) |  | 1(ref) |  |
| Bachelor/master degree | |  | 2.03 (0.90, 4.59) | 0.09 | 2.12 (0.90, 4.98) | 0.09 |
| **Years of work** | |  |  |  |  |  |
| 1-5 |  |  | 1(ref) |  | 1(ref) |  |
| 6-10 |  |  | 1.67 (0.72, 3.90) | 0.24 | 1.82 (0.76, 4.37) | 0.18 |
| ≥11 |  |  | 1.23 (0.46, 3.29) | 0.68 | 1.43 (0.51, 4.02) | 0.50 |
| **Employment form** | |  |  |  |  |  |
| Contract staff |  |  | 1(ref) |  | 1(ref) |  |
| Formal staff |  |  | 1.09 (0.57, 2.06) | 0.80 | 1.07 (0.54, 2.10) | 0.86 |
| **Monthly salary (CNY)** | |  |  |  |  |  |
| ＜ 5,000 |  |  | 1(ref) |  | 1(ref) |  |
| 5,000-10,000 |  |  | 1.14 (0.36, 3.68) | 0.82 | 1.21 (0.37, 3.98) | 0.76 |
| ＞10,000 |  |  | 2.31 (0.66, 8.05) | 0.19 | 2.62 (0.73, 9.37) | 0.14 |
| **Shift work** |  |  |  |  |  |  |
| No |  |  | 1(ref) |  | 1(ref) |  |
| Yes |  |  | 2.84 (1.02, 7.92) | 0.05 | 3.03 (1.07, 8.57) | 0.04 |
| **Delivery mode** |  |  |  |  |  |  |
| Vaginal delivery | |  |  |  | 1(ref) |  |
| Cesarean section | |  |  |  | 0.63 (0.31, 1.28) | 0.20 |
| **Labor pain experience** | |  |  |  |  |  |
| Yes |  |  |  |  | 1(ref) |  |
| No |  |  |  |  | 0.79 (0.32, 1.94) | 0.61 |
| **Perinatal depression** | |  |  |  |  |  |
| Yes |  |  |  |  | 1(ref) |  |
| No |  |  |  |  | 0.94 (0.51, 1.73) | 0.83 |
| **Children age (years)** | |  |  |  |  |  |
| Preschool (3-5) |  |  |  |  | 1(ref) |  |
| Infant (＜3) |  |  |  |  | 2.81 (1.34, 5.92) | 0.01 |
| School-age (≥6) |  |  |  |  | 1.18 (0.44, 3.16) | 0.74 |
