# Supplementary figures and images for: Midwife empathy and its association with the childbirth experience: a cross-sectional study
Source: BMC Pregnancy Childbirth. 2022 Dec 22;22:960. doi: 10.1186/s12884-022-05309-3 (PMC9774080; doi:10.1186/s12884-022-05309-3)

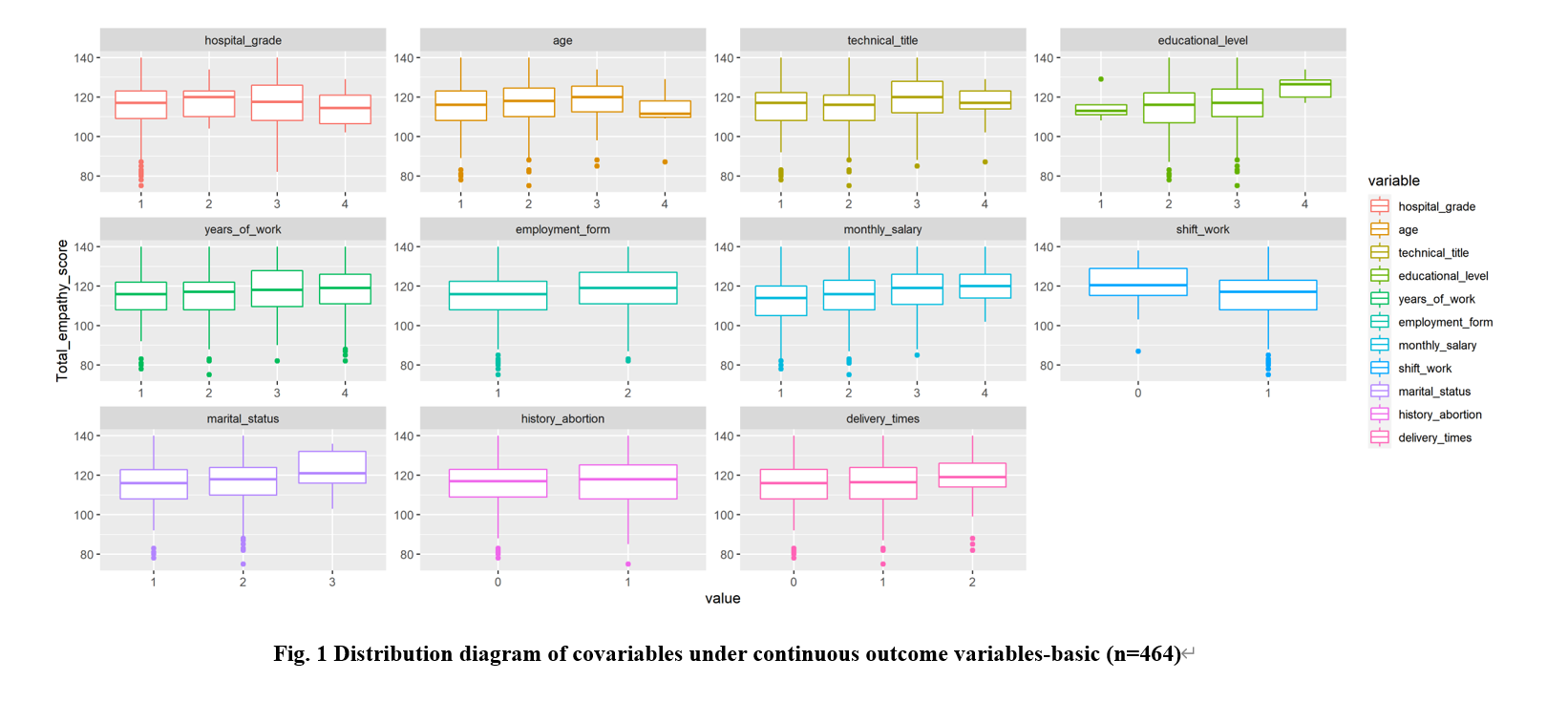

Supplement: Supplementary file 1 — Additional file 1. [file 12884_2022_5309_MOESM1_ESM.zip › Supplementary file/Distribution diagram of covariables under continuous outcome variables-basic (n=464).png]

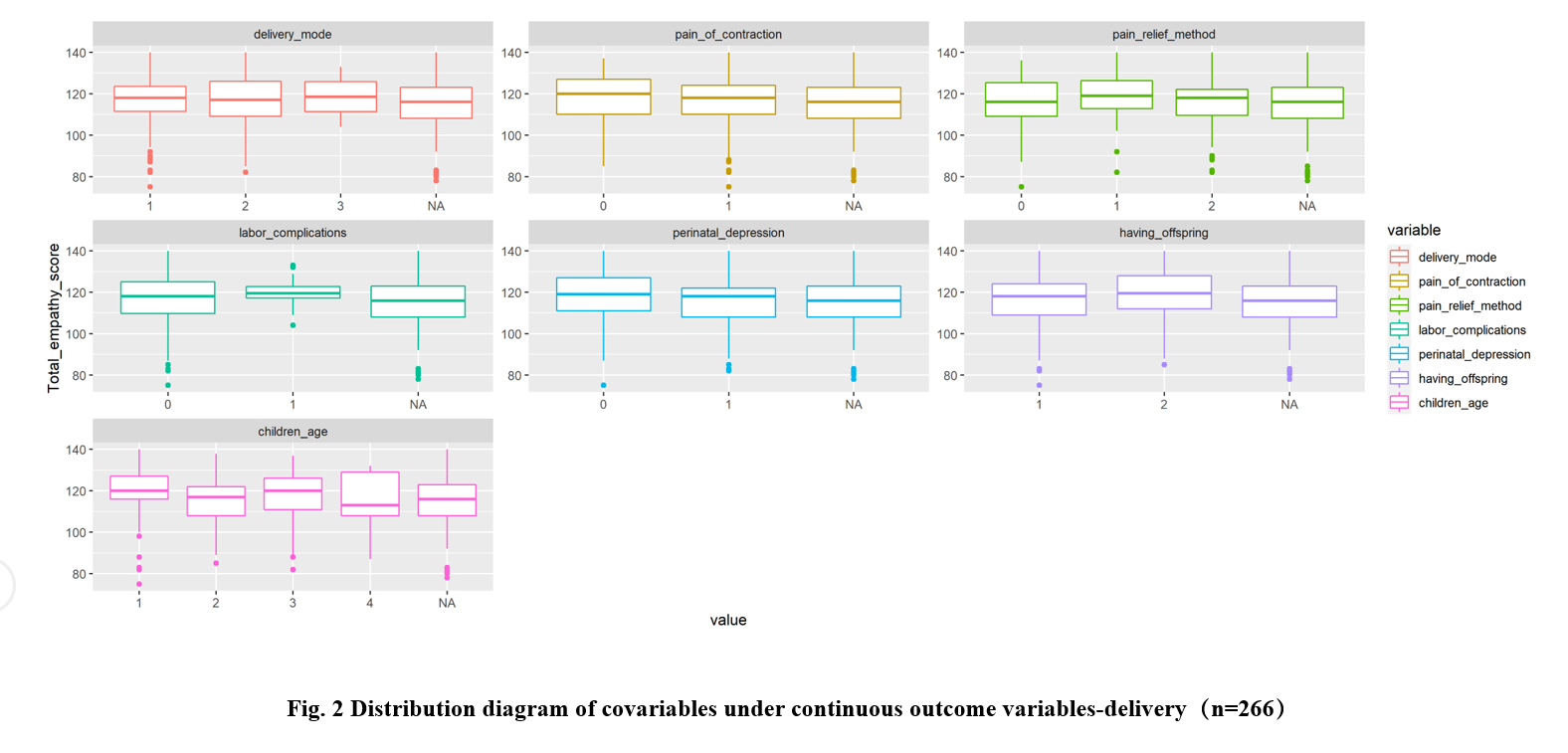

Supplement: Supplementary file 1 — Additional file 1. [file 12884_2022_5309_MOESM1_ESM.zip › Supplementary file/Distribution diagram of covariables under continuous outcome variables-deliveryú¿n=266ú⌐.png]
